# Learning health ‘safety’ within non-technical skills interprofessional simulation education: a qualitative study

**DOI:** 10.1080/10872981.2017.1272838

**Published:** 2017-01-19

**Authors:** Morris Gordon, Christopher W. R. Fell, Helen Box, Michael Farrell, Alison Stewart

**Affiliations:** ^a^ Department of Medical Education, Blackpool Victoria Hospital, Blackpool, UK; ^b^ School of Medicine and Dentistry, University of Central Lancashire, Preston, UK; ^c^ School of Psychology, University of Central Lancashire, Preston, UK

**Keywords:** Non-technical skills, intergroup contact anxiety, simulation training, human factors

## Abstract

**Background**: Healthcare increasingly recognises and focusses on the phenomena of ‘safe practice’ and ‘patient safety.’ Success with non-technical skills (NTS) training in other industries has led to widespread transposition to healthcare education, with communication and teamwork skills central to NTS frameworks.

**Objective:** This study set out to identify how the context of interprofessional simulation learning influences NTS acquisition and development of ‘safety’ amongst learners.

**Methods**: Participants receiving a non-technical skills (NTS) safety focussed training package were invited to take part in a focus group interview which set out to explore communication, teamwork, and the phenomenon of safety in the context of the learning experiences they had within the training programme. The analysis was aligned with a constructivist paradigm and took an interactive methodological approach. The analysis proceeded through three stages, consisting of open, axial, and selective coding, with constant comparisons taking place throughout each phase. Each stage provided categories that could be used to explore the themes of the data. Additionally, to ensure thematic saturation, transcripts of observed simulated learning encounters were then analysed.

**Results**: Six themes were established at the axial coding level, i.e., analytical skills, personal behaviours, communication, teamwork, context, and pedagogy. Underlying these themes, two principal concepts emerged, namely: intergroup contact anxiety – as both a result of and determinant of communication – and teamwork, both of which must be considered in relation to context. These concepts have subsequently been used to propose a framework for NTS learning.

**Conclusions**: This study highlights the role of intergroup contact anxiety and teamwork as factors in NTS behaviour and its dissipation through interprofessional simulation learning. Therefore, this should be a key consideration in NTS education. Future research is needed to consider the role of the affective non-technical attributes of intergroup contact anxiety and teamwork as focuses for education and determinants of safe behaviour.

**Abbreviations:** AUM: Anxiety/uncertainty management; NTS: Non-technical skills; TINSELS: Training in non-technical skills to enhance levels of medicines safety

## Introduction

Healthcare within the 21st century has focussed more than ever before on the phenomena of ‘safe practice’ and ‘patient safety’ [1,2]. To enable organisations to achieve appropriate delivery of healthcare within the context of these constructs, there has been much interest in the concept of human factors engineering to enhance safety; a branch of social science derived from psychological principles, and pioneered in the aviation industry. In examining the interactions between a workplace system and the people working within it, human factors engineering aims to improve workplace safety through optimisation of the environment itself – not the people working within the environment – in order to mitigate error [3]. It is noted by key workers in the field [4] that many educators within healthcare have reinterpreted human factors, focussing instead on the ‘human’ element. This focus is only a small part of human factors engineering which is best understood through the educational lens of non-technical skills (NTS) training. NTS has been previously defined as the cognitive and interpersonal skills that complement an individual’s professional and technical knowledge in the facilitation of effective delivery of a safe service [5]. NTS training within the aviation industry, also known as Cockpit Resource Management (CRM) [6], is ubiquitous; and whilst the theoretical underpinning of such work is often difficult to ascertain [7], such training is situated within an industrial context that is open and proactive in the reduction of risk and the improvement of safety.

In the light of these successfully integrated NTS programmes, it is no surprise that healthcare educators trying to foster ‘safe practice’ have looked to transpose these techniques [5]. NTS has been defined in the healthcare setting as ‘a set of social (communication and teamwork) and cognitive (analytical and personal behaviour) skills that support high quality, safe, effective, and efficient interprofessional care within the complex healthcare system’ [8]. Herein, the term interprofessional refers to a situation or environment in which practitioners of two or more disciplines work, or learn, interactively and collaboratively rather than in parallel [9]. However, there is a paucity of published works that are theoretically underpinned, or that consider a practicable and sustainable pedagogy, except for a broad consensus regarding the use of simulation. Instead, a focus on considering ‘whether’ NTS safety interventions work, rather than addressing the ‘how’ and the ‘why’ have left a clear gap in the evidence base, demonstrated within a recent systematic review [5]. More recent studies have sought to propose the conceptual and theoretical frameworks that influence NTS learning. One such model ‘describes the three areas that facilitate learning of non-technical skills to enhance safety in healthcare’ [10], these being: (1) core knowledge and skills, which contribute to and support NTS learning; (2) situated cognition, being the approach to learning that develops these skills; and (3) analytical skills, which integrate these elements to inform decision making. Within these areas, seven key elements that are cyclically linked are suggested, with (1) local Systems and technology, (2) Error awareness, (3) Communication, (4) Teamwork, and (5) Observation & experience informing (6) Risk assessment and (7) Situational awareness (SECTORS) that, in turn, lead to decision making. These studies have been grounded primarily in the published health education literature rather than prospective inquiry [11], and to date no works have attempted to reject or amend this model. Indeed, whilst this approach suggests the role of situated cognition in behaviour change, further understanding as to how learning can enhance NTS is required.

Core to all previously published NTS assessment frameworks (12–17), as well as the most current SECTORS model of NTS learning in health [10], are communication and teamwork skills considered from a psychosocial perspective. The frameworks support the deployment of simulation as a methodology to develop and demonstrate core NTS and develop safety [5]. However, clarity as to how these two skill sets are regulated through simulation learning is not addressed within these cited works [5,10,12–17]. Interprofessional simulation may provide an additional contextual facet to the learners’ understanding of NTS, introducing its own unique influence when compared against other educational methods. Investigation in this area will clarify how such skills can support ‘safety’ and in what manner simulation should be deployed to support the acquisition of NTS. The present study therefore set out to answer the research question: how does the context of interprofessional simulation learning influence NTS acquisition and development of ‘safety’ amongst learners?

## Methods

This study was completed within the wider context of the Training in Non-technical Skills to Enhance Levels of medicines Safety (TINSELS) programme [18]. This previously reported simulation-based interprofessional training programme was developed and piloted in Blackpool Victoria Hospital Simulation Unit, UK. The programme was designed using the SECTORS model to guide content and overall pedagogical direction, with the employment of complexity theory to support the application of simulation as a method in this context [19].

In brief, a three-session simulation-based intervention was produced: session one was a simulated ward encounter with multiple medicine-related activities, with immediate debriefing; session two included an extended debriefing and facilitated discussion with selected video extracts from the first scenario; and session three a ‘chamber of horrors’ where interprofessional teams identified potential sources of error on a simulated ward. Each session was completed in the simulation suite with six to nine participants and lasted approximately 90 minutes. After the completion of all elements of the programme, participants were invited to attend a separate set of focus groups. Being a method of choice in both exploratory and explanatory medical education research [20], focus groups were selected to best capture the richness of the human experience of NTS acquisition within the context of interprofessional simulation training. Recent precedents include explorations of ‘lived’ student experiences [21], documentation of their thoughts and feelings [22], and explanations of their behaviour [23]. Ethical approval was obtained for these focus groups as part of the wider approval for the project by the local Research and Development department as well as Health Education North West. The focus groups set out to explore communication, team working, and the phenomena of safety in the context of the learning experiences the learners had within the TINSELS programme. Participants were all learners who had completed the TINSELS training programme and were randomly assigned to focus groups in January 2015 for sessions that lasted 90 minutes each. A succinct semi-structured transcript was developed to facilitate discussions with regards to the phenomena of safety and the interprofessional learning environment.

The focus groups were recorded using a digital Dictaphone and, to ensure anonymity, were transcribed by a clinical administrator with no other involvement with the study. Transcripts were subsequently imported into NVivo version 10.0.638.0 SP6 (QSR International Pty Ltd, Melbourne, Australia) for analysis. The analysis was aligned with a constructivist paradigm [24], in that it was concerned with building a picture of how communication, teamwork, and the phenomenon of safety were experienced in the context of the interprofessional NTS simulation learning programme undertaken by participants. Accordingly, following the use of focus group interviews, an investigator-as-instrument type fieldwork method of inquiry, an inductive approach was taken to create a narrative output [25]. Whilst our hypothesis and use of existing models [10] to underpin the teaching programme formed a schemata for initial thematic development, we avoided making *a priori* conclusions [26].

The analysis proceeded through three stages consisting of open, axial, and selective coding with constant comparisons taking place throughout each phase [27]. This was done by two researchers, and any disagreement solved by reaching consensus with a third author. Each stage provided categories that could be used to explore the themes of the data. Additionally, to ensure thematic saturation and triangulation of data, transcripts of all the TINSELS simulated learning encounters were then analysed.

## Results

Acceptance of invitation was received from 12 participants, and two focus groups were completed. Participants included three junior doctors, one undergraduate medical student, three second-year undergraduate student nurses, three third-year undergraduate student nurses, two pharmacists, and one occupational therapist. The undergraduates represented two different universities. A total of 447 codes were extracted from the data. No new codes were obtained from the TINSELS training transcripts, thereby confirming appropriate theoretical saturation.

**Figure 1.**
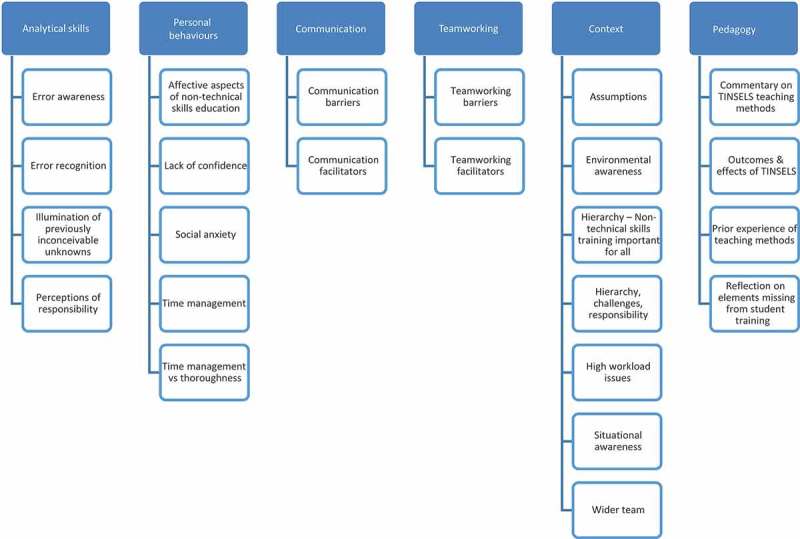
Open and axial coding themes.

Figure 1 shows the open and axial themes. In the open coding stage, 24 categories were developed. The next stage of the analysis established six comprehensive themes, i.e. Analytical skills, Personal behaviours, Communication, Teamwork, Context, and Pedagogy. See Table 1 for open and axial code descriptive specifications.

**Table 1.** Open and axial code descriptive specifications.

Level	Code	Descriptive specification
Axial	Analytical skills	Cognitive factors informing the ability to problem solve and decision make in a safety-focussed interprofessional healthcare environment
Open	Error awareness	Factors influencing the understanding of the occurrence of errors
Open	Error recognition	Factors influencing the perception rate of errors: insight into other roles, the contribution of interprofessional communication and teamwork
Open	Illumination of previously inconceivable unknowns	Gaining of knowledge that reveals a depth which was previously overlooked, the ‘why’ that reinforces the ‘how’
Open	Perceptions of responsibility	Commentaries on blame culture, elements of judgement in error risk and acceptance, the shift from blame culture to a wider team view
Axial	Personal behaviours	Performance influencing traits, qualities, and actions specific to the individual
Open	Affective aspects of NTS education	Emotional feedback evoked through direct experience of the interprofessional simulation and delivered content, and its impact on perceptions of patient safety
Open	Lack of confidence	Changes in behaviour or performance due to insufficient trust in one’s own abilities
Open	Social anxiety	Barriers (identifiers and reinforcers) and facilitators (in the workplace and through TINSELS training) related to preconceptual and experiential factors influencing interprofessional contact & communication
Open	Time management	The contributions of interprofessional communication to the efficient use of available time
Open	Time management vs. thoroughness	The interplay between perceived efficient use of available time and the meticulous completion of tasks in the interest of safety
Axial	Communication	Factors influencing the ability to effectively exchange information within an interprofessional team
Open	Communication barriers	Factors negatively influencing and reinforcing perceived difficulties in interprofessional communication, including: elements missing from previous training, lack of interprofessional experience, experiences of unapproachable staff, stigmas regarding other professions and junior staff
Open	Communication facilitators	Factors positively influencing interprofessional communication, including: insight into other roles, approachability, support, NTS training, and breaking down barriers through interprofessional simulation
Axial	Teamworking	Factors influencing the ability to perform as part of an effective, efficient, and safe interprofessional team
Open	Teamworking barriers	Factors negatively influencing effective teamwork, including: lack of insight into other roles, single-discipline focussed awareness and training, professionally compartmentalised workplaces, profession dependent treatment
Open	Teamworking facilitators	Factors positively influencing effective teamwork, including: insight into other disciplines’ roles and responsibilities, discovery of available support, and the increases in awareness and perspective gained through debrief and reflection as an interprofessional team
Axial	Context	Understanding of one’s own role when set against the individual roles of other healthcare professionals, the overall interprofessional team, factors of the workplace setting, and associated demands and goals
Open	Assumptions	Prior acceptance of things to be true without proof, thus increasing risk, along with changes in awareness of this and methods of mitigation
Open	Environmental awareness	Extending focus from being solely on the patient to include the surroundings
Open	Hierarchy: NTS training important for all	Conclusions that NTS training would be beneficial to colleagues from all levels of experience, authority, and disciplines involved in healthcare
Open	Hierarchy: challenges, responsibility	Implications for contesting the decisions or actions of seniors and other healthcare professionals due to patient safety being the duty of all
Open	High workload issues	Balancing NTS and patient safety against an over-abundance of tasks, information, and additional considerations
Open	Situational awareness	Identifying, processing, and suitably acting upon the critical components during an event
Open	Wider team	Insight acknowledging that effective interprofessional healthcare is bigger than an individual role or patient
Axial	Pedagogy	Comments regarding the methods and content of the course, along with its impact on ward work and comparisons to previously received training.
Open	Commentary on TINSELS teaching methods	Reflections on the relevance of simulation to placements; insight into performance and best practice provided through debrief; opportunities to change own practice through reflection; comparisons of video to real life; effectiveness of combining theoretical reading with practical experience; added value of experiential learning; and realism provided by interprofessional simulation.
Open	Outcomes & effects of TINSELS	Changes in behaviours and performance implemented in ward work subsequent to the course, including: awareness, challenging, error recognition, interprofessional communication, peer mentoring, preparation, and reflection.
Open	Prior experience of teaching methods	Reflections on lack of previous opportunities to undertake simulation for some disciplines, and interprofessional simulation for all disciplines.
Open	Reflection on elements missing from student training	Theoretical over practical experiences until qualified, lack of opportunity for practical experiences to reflect upon, intra-professional over interprofessional focus, and overlooking of precursory sequence to errors.

### Analytical skills

Analytical skills encompassed the existing NTS learning element of error awareness, but the data revealed in greater depth how this awareness informs and impacts upon behaviour, opening up a new conceptual understanding within learners:

‘Whenever I go onto Geriatrics [ward] after the teaching, I imagine multiple things all written up incorrectly. I am just like ’in‘ the scenarios that we did.’

Previously, the role of error awareness as a determinant of a professional’s taking of personal responsibility has been described [28] and linked to agency theory [29], with professionals at risk of becoming task-focussed and thus shirking professional responsibility. This data explored the concept of responsibility and the interplay between the individual and team in the context of ‘blame’ as a construct within ‘safe practice’:

‘Through our training it’s been drummed into us, that if you make a mistake … then it is your fault. I don’t think we have ever been told if you did make an error you look at the sequence, you see why.’

‘The course has taught me that you can’t blame an individual, it just makes you realise the bigger picture and how many things could have happened to lead up to that point, and I have learned that shared responsibility is part of error prevention in a shared [working] environment’

### Personal behaviours

Personal behaviours described a set of factors that have not been recognised as determinants of NTS learning in the previous model [5]. For example, it included the affective aspect of NTS learning and how this was seen by learners as causing personal emotional impact:

‘It still does put that worry across you, where you think, ’oh my god, what if that had not been a simulation’, what if we had been in a busy ward environment.

I know it is sim … but you still had that feeling and you learn from that.’

A key theme that emerged was that of social anxiety, particularly in the context of interprofessional communication when a variety of factors came into play, such as hierarchies of responsibility, approachability of colleagues from other groups, and pre-conceptions of interprofessional communication.

‘if they know the doctors very well… they will want to speak to that doctor. However if they are new to the ward then they will ask their [own] senior they know.’

Conversely, several factors were observed that dissipated such social anxiety, including the underlying patient focussed directive to challenge professionals from other groups, and efforts to break down perceived barriers between their professional group and others:

‘It will still be difficult to challenge a senior medic saying that I think the course has made me realise that that is the right thing to do and it’s important for patients’ safety, even if you’re not sure’

‘I have not actually communicated to any of the other [professional] staff; But on me doing the course, the importance of speaking to the others in the team was now there.’

One of the junior doctors specifically mentioned a change in communication with other professionals that has resulted from NTS learning:

‘Now every time I prescribe something on the ward round I physically go up to the nurse and just say ’oh look we have just changed this medication‘ or ’were prescribing this‘ … even if it means I am a little bit late to see the next patient’

Finally, the issue of balancing tasks and workload in a manner that promotes ‘safe practice’ was considered. A prime example is in shifting from a position of taking one’s time as a negative, to being considered a behaviour that promotes safety:

‘It’s made me feel less, what’s the word, less ’bothered‘ if you will, with what they are thinking because I know I am doing it for a purpose.’

### Communication and teamwork

Communication and teamwork were both identified as being both barriers to and enablers of ‘safe practice.’ Of note was the underpinning concept of the participants’ own professional group versus other professional groups influencing these themes:

‘on some of the wards I have been on, I think I have tried to speak and talk to someone and it’s like, ’I don’t care, I’m higher than you so I am not going to listen.‘ I think it depends where you work and who you are working with and if you can consult things with them.’

‘I have never really seen [my mentors] communicate much with pharmacists or encouraging anybody to communicate with the pharmacists’

‘I think I would go to a nurse over a doctor because you are more comfortable around nursing staff because that’s what we are trained to do’

A particularly interesting view was expressed from a student nurse regarding the breakdown of these perceived barriers to intergroup communication:

‘I think some of the nurses on the ward will go to a sister and ask them for advice before they go to a doctor. Whereas I now would go to a doctor before going to a senior nurse … if they are going to go and ask the doctor anyway’

A medical student also expressed that intergroup communication is important to explore differing yet equally valuable viewpoints:

‘As a group you get to see what other people think of the situation … so you understand basically what everyone else feels in terms of their role and what also what they think and, what you could have done sort of thing.’

### Context

Context was a theme that overlapped and influenced all of the other elements, as may be expected when considering a human factors model of safety. Whilst environment and systems were discussed, as has been common in previous investigations of safety learning [28], it was often through the perspective of the individuals working within these systems. Once again the issue of group dynamics was a recurring element:

‘to just assume that somebody from another [group] knows what I actually do, … not everyone actually knows what we are supposed to be doing’

‘I just think it is important that we do come together every day in our [work environments], like we did in the scenarios … because we all noticed totally different things to one another’

### Pedagogy

Finally, several codes clearly related to pedagogy. For instance, the interprofessional authenticity of the approach was identified:

‘I have done sim in the past … but these sessions … it’s a lot more real. You get the experience from all different disciplines rather than just your own … you get the wider’

‘When we have done sim at uni … it’s just been our group so you do feel more comfortable as you know the sim, because you know each other but then doing this here you make up other disciplines so you don’t know what could happen on the ward so it does make it more real.’

This was also identified in the context of debrief and receiving feedback from interprofessional peers:

‘You don’t realise until … you get feedback from other health care professionals on what job you have just all done it kind of opens your eyes a little bit.’

‘it gives us the chance to talk to each other and break down the barriers between the different [groups] as well, because we have all had to communicate together.’

When reflecting on this, one of the doctors highlighted how this programme of simulated training filled a gap in terms of actual training in interprofessional working. Additionally, a student nurse highlighted the hypocrisy in being taught about NTS and safety in a purely theoretical manner whilst not acquiring experience until there is the potential to harm patients:

‘As a medic you go through medical school being told we really need to learn how to work effectively amongst the team, but we have no real involvement with them.’

‘In lectures and stuff, we get warned a lot about the risks and what can happen but as a student you're pretty much always working with your mentor and it is not until you’re going to qualify that this could happen so you don’t really think of the risks too much because you are covered’

In moving to the final selective coding level of analysis, two elements within the axial coding appear to be key in determining and regulating NTS learning. Firstly, context considered from a human factors perspective. Previously the NTS focus has been on specific systems and processes, with defined behavioural outcomes, such as communication skills. A human factors context perspective is a more abstract view that considers contextual hierarchies, skill and professional mix, workload issues, and likely environmental assumptions. Thereby, the concept of situational awareness is redefined at a team level. Previous works concentrate at a task or patient focussed level (12–17), which is paradoxically at odds with how humans interact within their environment [6].

Secondly, group interactions were highlighted throughout, in particular with the emergence of the individual’s ‘self-concept’, as informed by their social identity. This self-conception arises from the ethnocentrism of group membership, wherein the participants have identified themselves with their own perceived group (ingroup) over another group (outgroup) [30]. Within the context of this ingroup/outgroup paradigm, intergroup contact can be affected by unfavourable attitudes towards an outgroup, fostered by prevailing social ideologies, and perpetuated by a dearth of opportunities to disconfirm or positively revise attitudes [31]. Within an interprofessional workplace, practitioners of one’s own profession may be considered the ingroup, whereas practitioners of another occupation may be considered the outgroup. This concept goes beyond the existing constructs of communication and teamwork within NTS learning or the concept of ‘personal behaviours’ and, what is more, describes an archetype of affective attributes, overlapping and impacting social skills, and influenced by context.

**Figure 2.**
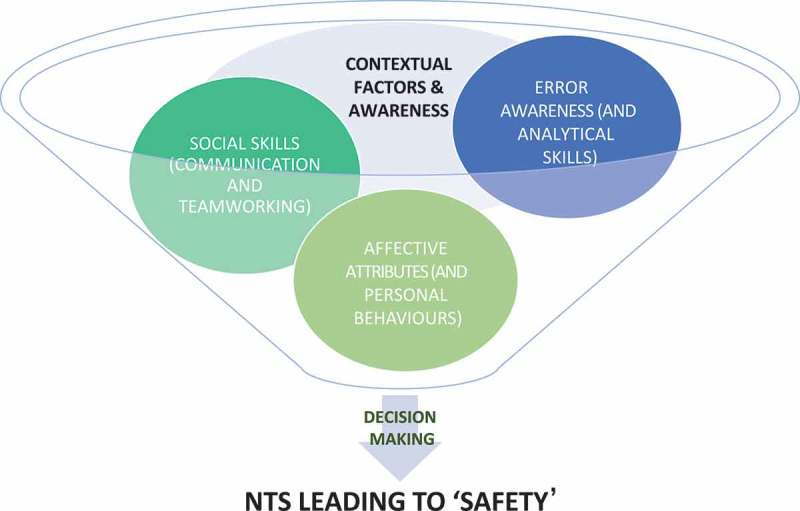
Model of NTS learning synthesised.

These two concepts were bound by the authors in a newly refined model of NTS (Figure 2), which was influenced by the previous framework, but grounded in the data.

## Discussion

With the increasing deployment of interprofessional simulation education that aims to achieve NTS learning, the results of this study allow educators to better understand how the individual elements that are routinely described within the NTS paradigm (12–17) are interrelated and can be addressed within educational interventions. Key to this is the emergence of a concept of social identity and how individuals interact differently between the members of the ingroup and outgroup. According to Allport’s contact hypothesis [32], intergroup interactions result in positive outcomes in situations where four conditions exist: (1) equal status between groups, (2) common goals, (3) intergroup cooperation, and (4) the support of authority. Expanding on this, intergroup contact theory states that even where these conditions exist, other factors such as emotional and behavioural dispositions towards the outgroup are critical to ensuring positive outcomes in that they alter our perceptions of threat and associated anxieties [33]. Four sources of such threat and anxiety are described by the integrated threat model of intergroup contact, two of which are relevant in this setting, namely intergroup anxiety, being a threat to the self through embarrassment or rejection, for example; and negative stereotypes, being the anticipation of intergroup anxiety [34]. We propose that our results demonstrate evidence of both of these theoretical elements.

Given the existence within the data of what can clearly be described as intergroup contact anxiety being a form of social anxiety specific to contact situations between groups, anxiety/uncertainty management (AUM) theory can be applied [35]. This theory postulates that effective intergroup communication may be achieved through the management of such anxieties. Herein, effective intergroup communication occurs between two anxiety thresholds corresponding to the maximum and minimum levels of anxiety that a person can experience, while still being comfortable with interaction. AUM theory contains a number of axioms related to situational processes which may be applied to the professional workplace, especially with regard to the reduction of errors through the use of NTS. These axioms reason that an increased complexity in the expected set of coherent events (scripts), an increased cooperative goal structure, and increased institutional and normative support for such interactions may reduce anxiety. Furthermore, in novel workplace relationships, AUM theory proposes that increased interdependence may be similarly beneficial [36]. Thus, promoting effective intergroup communication and teamwork within NTS training should be achieved through consideration of these intergroup contact anxiety theoretical elements.

Central to this paradigm are the interactions within authentic interprofessional learner groups. Whilst these may seem like an apparent requirement of such work, it is common for team-work focussed learning in health education to use single professional learner groups [37], whereas interprofessional teams undertaking learning in a simulated environment offer an exposure-based pedagogical option. This method can foster the development of the interdependence, increased complexity of scripts, increased cooperative goal structure, and increased normative support for such interactions, all described as addressing intergroup contact anxiety. Conversely, the results strongly question the benefit of simulation training to achieve NTS competencies if this is based within homogenous professional groups, although this is very common [5].

When considering these results, several limitations must also be noted: firstly, contextual issues including studying a single hospital setting and with learners from two undergraduate institutions; secondly, methodological limitations of the focus group method must be noted; thirdly, given the central role of social anxiety, it is difficult to ascertain how much of this affect could be related to innate levels within our participants; and finally, the participants were from a larger study investigating a specific simulation-based NTS intervention, so this context must be considered as a potential source of bias. When considered together, these limitations may impact the wider generalisability of the results, requiring further verification work to take place.

Additionally, future works are needed to investigate the role of intergroup contact anxiety as both a determinant of error promoting behaviour within a healthcare setting and as a focus for NTS educational outcomes. Related to the latter point, clarification is needed as to the impact of the use of an interprofessional simulation model in the acquisition and retention of NTS compared with similar techniques using homogenous professional groups.

## Conclusions

This study has highlighted the role of intergroup contact anxiety as a factor in NTS behaviour and that this is impacted by learning within the interprofessional simulation environment. Authentic interprofessional learner groups undertaking simulation-based training support the development of NTS that can dissipate intergroup contact anxiety. Therefore, this should be a key consideration in NTS education. Future research is needed to consider the role of these affective attributes as a focus for education, and in determining safe behaviour.
